# The role of actin in capacitation-related signaling: an in silico and in vitro study

**DOI:** 10.1186/1752-0509-5-47

**Published:** 2011-03-30

**Authors:** Nicola Bernabò, Paolo Berardinelli, Annunziata Mauro, Valentina Russo, Pia Lucidi, Mauro Mattioli, Barbara Barboni

**Affiliations:** 1 Department of Comparative Biomedical Sciences, University of Teramo, 64100 Teramo, Italy

## Abstract

**Background:**

The signalling cascades involved in many biological processes require the coordination of different subcellular districts. It is the case of the pathways involved in spermatozoa acquisition of fertilizing ability (the so called "capacitation"). In the present work the coordination of subcellular signalling, during the boar sperm capacitation, was studied by a computational and experimental approach. As first the biological network representing all the molecular interactions involved in capacitation was build and analyzed, then, an experimental set up was carried out to confirm the computational model-based prediction.

**Results:**

The analysis of computational model pointed out that the "actin polymerization" node had some important and unique features:

- it is one of the most connected nodes,

- it links in a specific manner all the intracellular compartments,

- its removal from the network did not affect the global network topology but caused the loss of five important nodes (and among them the "plasma membrane" and "outer acrosome membrane" fusion).

Thus, it was suggested that actin polymerization could be involved in the signaling coordination of different subcellular districts, and that its functional ablation could compromise spermatozoa ability to complete the capacitation (while the main signaling pathway remained unaffected). The experiments, carried out inhibiting the actin polymerization in capacitating boar spermatozoa by the administration of cytocalasin D (CD), demonstrated that the CD treatment inhibited spermatozoa ability to reach the full fertilizing ability, while, the examined signaling pathways (membrane acquisition of chlortetracicline pattern C, protein tyrosine phosphorylation, phospholipase C-γ1 relocalization, intracellular calcium response to zonae pellucidae) remained effective, thus, confirming the model-based hypothesis.

**Conclusions:**

The model based-hypothesis was confirmed by the reported data obtained with the in vitro experiments, which strengthen the idea that the actin cytoskeleton is not only a mechanical support for the cell, but that it exerts a key role in signaling during the sperm capacitation.

## Background

One of the most important events in the history of life was the eukaryotes evolution that started with the onset of intracellular compartmentalization, approximately 1.6-2.1 billion years ago [1]. This fundamental step had enormous consequences on cell organization and function. In fact, in one hand, the appearance of specialized districts within the cytoplasm increased the efficiency of metabolic and signaling pathways, in the other hand, cell complexity increased. In fact, the development of organelles determined the existence of specific subcellular microenvironments characterized by different biochemical properties. Thus, the cell complexity arose either by the large number of molecular components involved in signal transduction and by the reciprocal connections and spatial relationship existing among them [2]. In addition, the spatial segregation of molecules and chemical reactions allowed that the same molecule could be responsible of very different signals. "We already have signaling "wires" distinguished by the identity of the molecules in the pathways. Compartmentalization duplicates these existing wires and separates them in space. This multiplies the number of signals they can carry" [2]. This increasing complexity imposes new biological problems as the emergence of new proprieties of cellular systems and the need of the presence of a/some conductor/s which harmonize the function of subcellular compartments. Recently the interpretation of the molecular mechanisms involved in cell signaling is made possible by new computational approaches that allow to modellize cellular functions as a network of integrated and coordinated signals operating within subcellular districts interconnected to each other [3,4]. One clear example is represented by sperm cells, whose biochemical mechanisms leading to capacitation during the post-ejaculatory life, were recently described by using the biological networks formalism [5]. The spermatozoa offer several advantages to adopt this kind of approach since:

- the main molecular events occurring during sperm capacitation are largely studied for biological and clinical reasons;

- their maturation can be experimentally reproduced under in vitro condition and assessed by measuring their ability to undergo acrosomal reaction (AR) after zonae pellucidae stimulation (ZP) or to in vitro fertilize the oocytes (IVF);

- their molecular composition is stable since the nucleus of the male germ cells is transcriptionally silent except for the mitochondrial protein translation of nuclear-encoded proteins [6].

Starting from these premises, the present work has been carried out, first, to study, using a computational approach, the mechanisms involved in the coordination of the molecular events that take place in the different subcellular districts during the post-ejaculatory spermatozoa maturation, the capacitation. Then, an experimental set-up was developed to confirm the model-based prediction. To this aim the events potentially acting as coordinators were inhibited by specific drugs during the spermatozoa incubation under capacitating conditions. The effects of this inhibition were assessed on functional spermatozoa status (ZP-induced AR), and on the major cellular events involved in the acquisition of full fertilizing ability (membrane acquisition of chlortetracycline pattern C, protein tyrosine phosphorylation, phospholipase C-γ1 relocalization, intracellular calcium response to ZP).

## Results

### Computational model of spermatozoa capacitation

The main topological parameters of the network representing the capacitation are shown in Table 1. The distribution of node linkages followed a power law, represented by the generic equation:

were

and

**Table 1:** Main topological parameters of capacitation network

Parameter	Value
N° nodes	153
N° edges	204
Clustering coefficient	0.056
Diameter	12
Averaged n° neighbours	2.654
Char. path length	4.995

The number of nodes represent the total number of molecules involved, the number of edges represents the total number of interaction found, the clustering coefficient is calculated as *C*I = 2*n*I/*k*(*k*-1), where *n*I is the number of links connecting the *k*I neighbours of node I to each other, the network diameter is the largest distance between two nodes, the Averaged n° neighbours represent the mean number of connection of each node, the Char. path length gives the expected distance between two connected nodes.

The r, R^2 ^coefficients were, respectively, 0.825 and 0.849.

The clustering coefficient distribution does not follow a power law, thus, the results of power law fitting of clustering coefficient distribution were: r = 0.120; R^2 ^= 0.232.

The most connected nodes are showed in Table 2.

**Table 2:** Most connected nodes (the hubs) of capacitation network

Node	Number of links
[Ca^2+^]_i_	28
ATP	15
Tyr phosphorylation	13
PKA	9
ADP	8
PLD1	8
NADH	8
Actin polymerization	8

**Figure 1:**
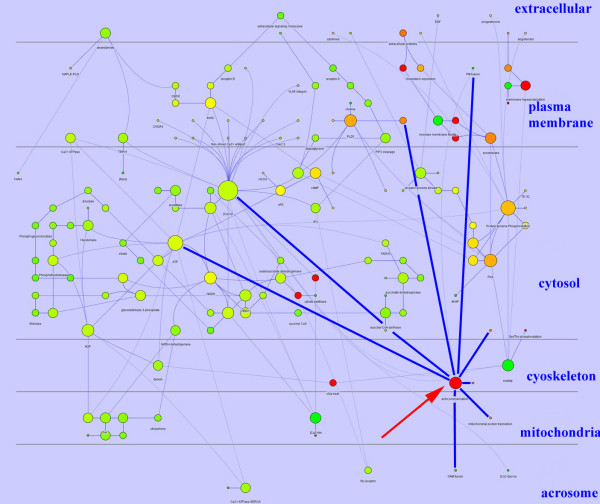
**Diagram showing the structure of the boar spermatozoa capacitation network and the subcellular localization of nodes**. The node size was proportional to the connection number and the node color gradient was dependent from the closeness centrality. This parameter is computed as: C_c_(*n*) = 1/avg(L(*n,m*)), were L (n,m) is the length of the shortest path between two nodes *n *and *m*. The closeness centrality of each node ranges from 0 (red) to 1 (green) and it is a measure of how fast the information spreads from a given node to the other nodes. The actn polymerization node is indicated by the red arrow, its links by blue line. All the nodes were localized in their specific subcellular domain (Cerebral V.2).

The analysis of the network topology revealed that only tree nodes bound all the subcellular compartments involved in post-ejaculatory signalling: [Ca^2+^]_i_, ATP, and "actin polymerization". [Ca^2+^]_i _and ATP are the most linked nodes and bound about one third of the nodes (48/151). More interesting is the behaviour of the "actin polymerization" node, represented in Figure 1, that links a smaller number of nodes (8) located in all the subcellular compartments (membrane, cytosol, cytoskeleton, mitochondria, acrosome).

**Figure 2:**
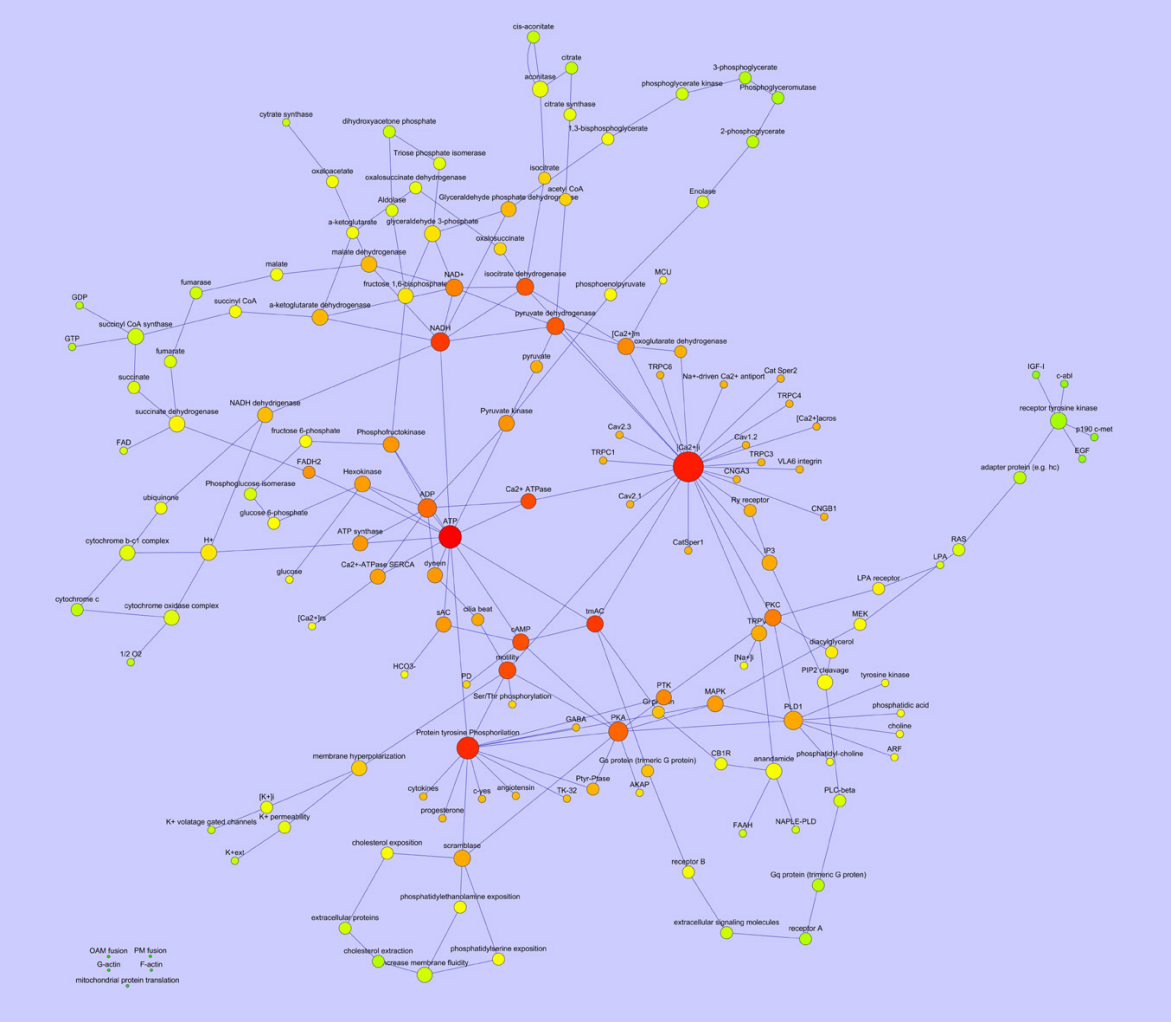
**Diagram showing the structure of the boar spermatozoa capacitation network after "actin polymerization" node removal**. The node size was proportional to the connection number and the node color gradient was dependent from the closeness centrality. This parameter is computed as: C_c_(*n*) = 1/avg(L(*n,m*)), were L (n,m) is the length of the shortest path between two nodes *n *and *m*. The closeness centrality of each node ranges from 0 (red) to 1 (green) and it is a measure of how fast the information spreads from a given node to the other nodes. The spatial network arrangement was obtained by using the Cytoscape Spring-embedded Layout (see the text for explanation).

The removal of the "actin polymerization" node from the capacitation network had modest consequences on the network topological parameter (as shown in Table 3), but caused the loss of 5 nodes: OAM fusion, PM fusion, G-actin, F-actin, mitochondrial protein translation (see Figure 2).

**Table 3:** Main topological parameters of capacitation network after "actin polymerization" node removal

Parameter	Value
N° nodes	152
N° edges	196
Clustering coefficient	0.056
Diameter	12
Averaged n° neighbours	2.566
Char. path length	6.071
Degree distribution	b = -1.563
	r = 0.809 R^2 ^= 0.898

The number of nodes represent the total number of molecules involved, the number of edges represents the total number of interaction found, the clustering coefficient is calculated as *C*I = 2*n*I/*k*(*k*-1), where *n*I is the number of links connecting the *k*I neighbours of node I to each other, the network diameter is the largest distance between two nodes, the Averaged n° neighbours represent the mean number of connection of each node, the Char. path length gives the expected distance between two connected nodes.

### Phalloidin staining

**Figure 3:**
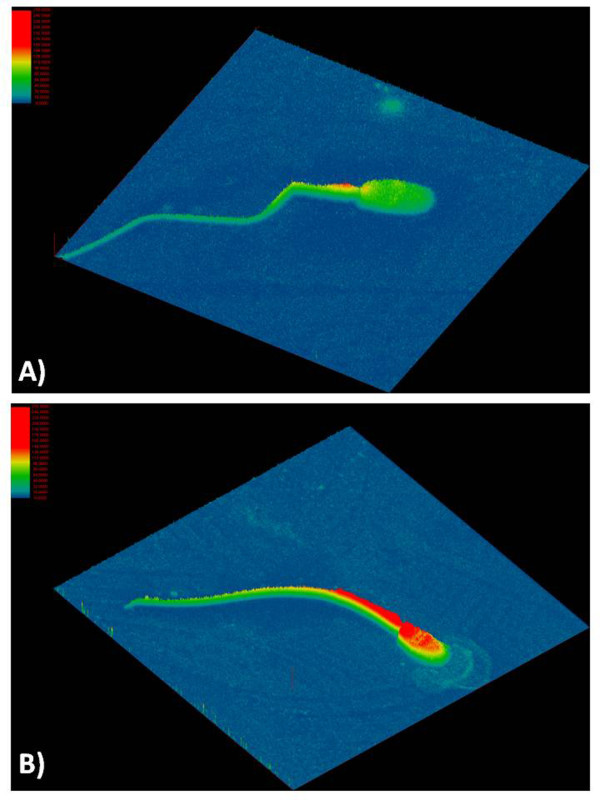
**Confocal image representing actin localization in ejaculated and in capacitated spermatozoa**. Confocal image representative of TRITC-phalloidin distribution on freshly ejaculated spermatozoon showing a faint fluorescence emission over the midpiece and post-acrosomal region (pattern A; panel A) and of incubated spermatozoon, displaying high fluorescence emission over the whole head (pattern B; panel B).

The distribution of the fluorescence within the spermatozoa was assessed, allowing the identification of two different patterns:

- pattern A) low actin polymeriziation: the phalloidin fluorescence emission is low and is mainly localized over the midpiece and the post acrosomial region (Figure 3A);

- pattern B) high actin polymerization: the phalloidin fluorescence emission is high and localized over the midpiece and the whole sperm head (Figure 3B).

The majority of freshly ejaculated spermatozoa localized a faint fluorescence emission in the midpiece and in the post acrosomal region (pattern A in 99 of 108 analysed spermatozoa). After 4 h of incubation under control conditions, the majority of spermatozoa increased their content of F-actin that resulted to be localized over sperm head (pattern B in 99 of 112 analysed spermatozoa; p < 0.01 vs. T0). The CD treated spermatozoa, displayed a fluorescence signal similar to that recorded at T0 (pattern A in 96 of 107 analysed spermatozoa; p > 0.05 vs. T0 and p < 0.01 vs. CTR).

### ZP-induced AR

**Figure 4:**
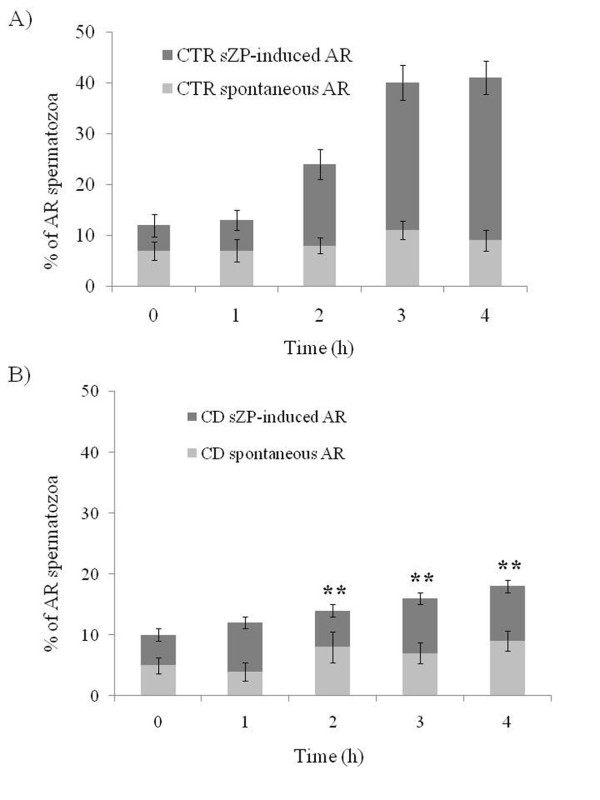
**Histogram representing the percentage of spermatozoa undergoing spontaneous and sZP-induced AR in CTR (A) and CD (B) treated spermatozoa**. Histograms showing the percentage of spermatozoa undergoing spontaneous (light gray) or sZP-induced (dark gray) AR in spermatozoa incubated under control conditions (panel A) or in the presence of CD (panel B). All the values are represented as mean ± SD. ** = p < 0.01 vs. CTR

The percentage of acrosome reacted spermatozoa recorded during incubation in control condition ranged from about 5% at the beginning of incubation to about 10% after 4 h. The sZP addition to the samples made possible to quantify the percentage of the spermatozoa responding to the physiological stimulus with the exocytosis of the acrosomal content (i.e. the cells which completed the capacitation process), which after the 4 h incubation reached about the 30% (Figure 4A). The CD treated spermatozoa showed an incidence of spontaneous AR similar to that recorded in the control samples during all the 4 h of incubation (p > 0.05), instead, the sZP addition promoted the AR in a markedly reduced percentage of cells (p < 0.01 vs. CTR), see Figure 4B.

### Chlortetracycline staining

The spermatozoa incubated under control conditions, at the beginning of incubation, showed three different patterns of chlortetracycline (CTC) stain in agreement with Mattioli et al. [7]:

- about 80% of spermatozoa displayed a faint fluorescence uniformly distributed over the head (pattern A);

- about the 10% spermatozoa displayed the fluorescence concentrated in the post-acrosomal region (pattern B);

- about the 10% displayed the fluorescence concentrated in the acrosome (pattern C): these spermatozoa, in keeping with Mattioli et al. [7], were considered to have completed the capacitation-related membrane reorganization.

**Figure 5:**
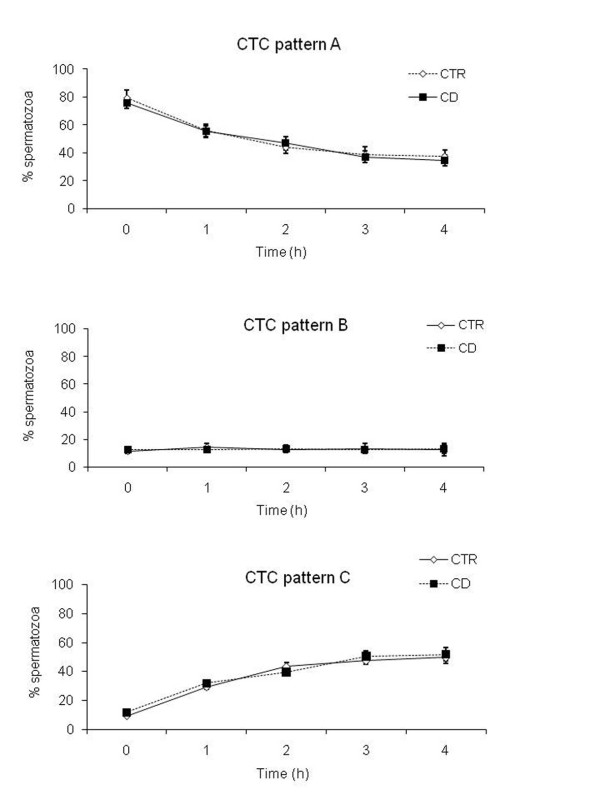
**Kinetic of acquisition of CTC staining pattern A, B and C in CTR and CD treated spermatozoa**. Graphic showing the percentage of spermatozoa displaying the CTC pattern A, B and C, during the 4 h of incubation under control conditions (dark continue line) or in the presence of CD (dark dot line). All the values are represented as mean ± SD.

After 4 h of incubation the percentage of the three patterns was markedly different: pattern A decreased to about 40%, pattern B remained at about 10%, while, pattern C reached 50%. As evident in Figure 5, the treatment with CD did not affect significantly the percentage of spermatozoa showing the different CTC stain patterns.

### Tyrosine phosphorylation

**Figure 6:**
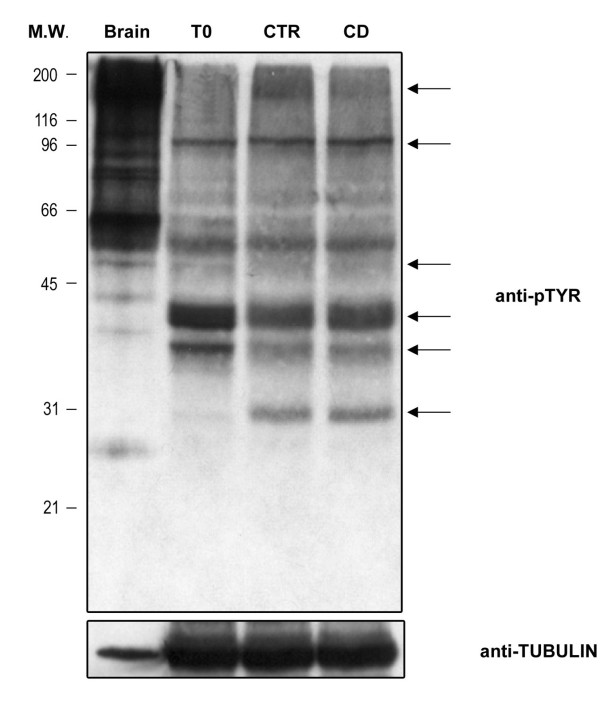
**Tyrosine phosphorolation pattern in ejaculated and in capacitated spermatozoa**. Western Blot analysis of tyrosine phosphorylation pattern in total lysate of freshly ejaculated male gametes (T0) or in spermatozoa incubated under control condition (CTR) or in the presence of CD (CD). The arrows indicate the capacitation-related changes in P-Tyr. Brain proteins were used as positive control. The filter was normalized on the Tubulin expression. The data shown were representative of three independent experiments.

The tyrosine phosphorilation pattern of freshly ejaculated male gametes (T0), or of spermatozoa incubated under control condition (CTR), or in the presence of CD was assessed. As summarized in Figure 6, after the incubation the tyrosine phosphorylation pattern changed regardless of the cultural condition adopted (CTR or CD).

### Phospholipase C-γ1 relocalization

**Figure 7:**
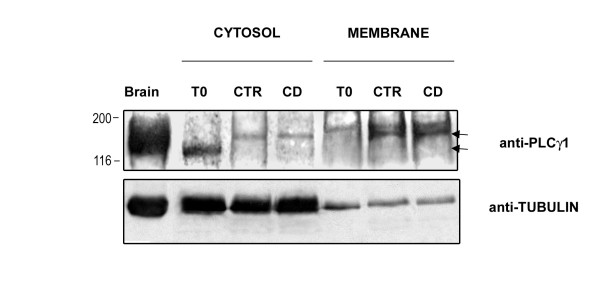
**Capacitation-dependent PLCγ1 relocalization**. Western Blot analysis of PLCγ1 localization in cytosolic and membrane fractions of freshly ejaculated male gametes (T0) or in spermatozoa incubated under control condition (CTR) or in the presence of CD (CD). The data showing the capacitation-dependent translocation of PLC-γ1 (arrows) from cytosol to membrane. Brain proteins were used as positive control. The filter was normalized on Tubulin expression. The data shown were representative of four independent experiments.

Phospholipase C-γ1 (PLC-γ1) protein expression studied with Western Blotting revealed that this protein modified its localization during the interval of culture. In fact, as shown in Figure 7, uncapacitated sperm cells (T0) strongly expressed PLC-γ1 within the cytoplasm, while, the incubation caused its translocation into the membrane compartment both in CTR and in CD samples.

**Figure 8:**
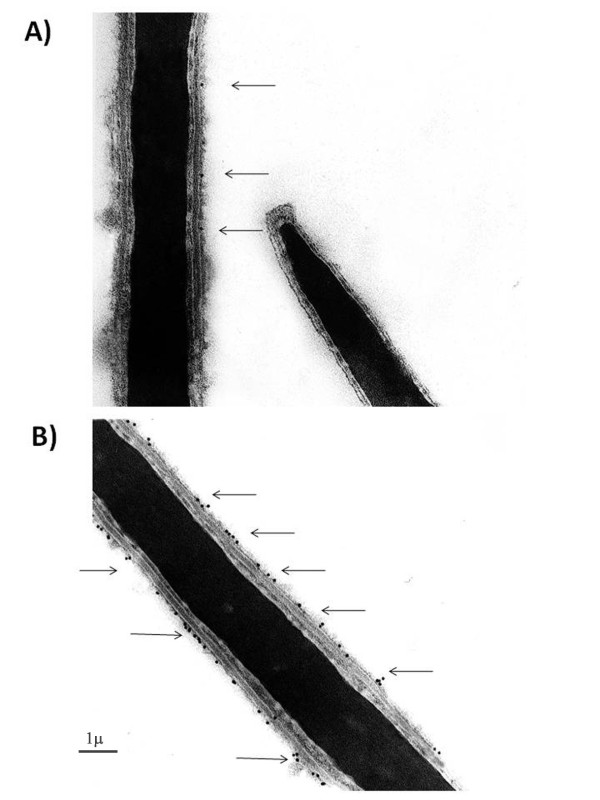
**Transmission electron microscopy pictures demonstrating the topography of capacitation-dependent PLCγ1 relocalization**. Transmission electron microscopy pictures (30.000X) showing: panel A): ejaculated spermatozoon displaying few gold particles (arrows) indicative of a low localization of PLCγ1 protein over cell membranes. panel B): in vitro capacitated spermatozoon incubated in the presence of CD that localized on its membrane several gold particles (arrows).

Ultrastructural analysis performed under pre-embedding condition, confirmed the membrane traslocation of PLC-γ1 in incubated spermatozoa maintained either in the presence or absence of CD, confirming the Western Blot analysis. In fact, uncapacitated spermatozoa displayed very few gold particles, indicative of PLC-γ1, on their membranes (Figure 8A), while, in *in vitro *incubated cells, independently from the used treatment, the number of gold particles localizing the protein on the membrane notably grew (Figure 8B).

### ZP-induced intracellular calcium concentration rise

**Figure 9:**
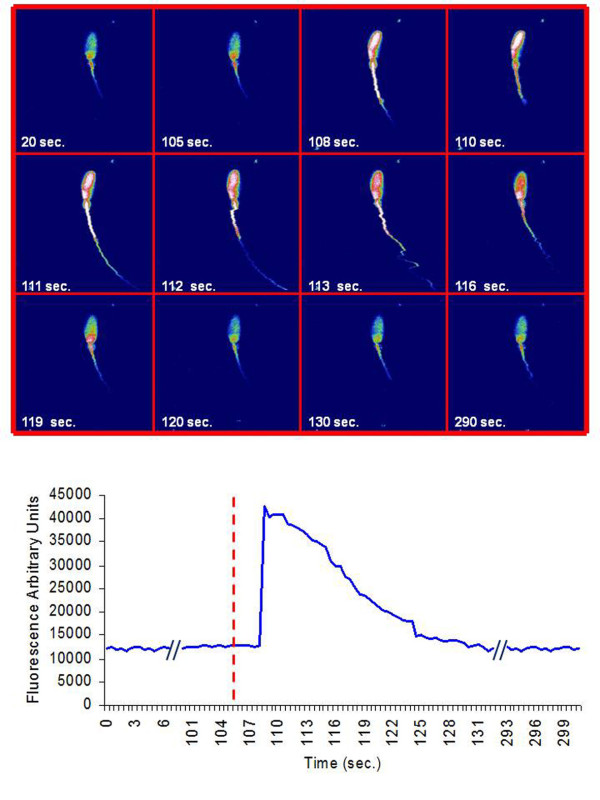
**An example of confocal image gallery of CD treated spermatozoa loaded with Fluo-3AM exposed to sZP**. An example of confocal image gallery of CD treated spermatozoa loaded with Fluo-3AM exposed to sZP (vertical red line). Notice that the rise in the [Ca^2+^]_i _in spermatozoon is evident after <10 sec. from sZP addition, whereas return on the baseline occurs after about 30 sec.

The coincubation with the sZP in spermatozoa capacitated under control conditions caused an evident [Ca^2+^]_i _elevation in the 36% of sperm cells (27 of 75 examined cells): the intracellular Ca^2+ ^peaked in 1-2 sec and lasted 20-40 sec before [Ca^2+^]_i _returning at a basal level. In the presence of CD the ability and the kinetic to respond with a [Ca^2+^]_i _rise to the sZP coincubation (Figure 9) was maintained in the 38% (32 of 83 examined cells) of spermatozoa (p > 0.05 vs. CTR).

## Discussion

Aim of this work was to study the molecular event/s involved in the coordination of the biochemistry of the subcellular compartments during spermatozoa acquisition of fertilizing ability (the so called capacitation) using a predictive computational modelisation and the in vitro experimental approach as a confirmation. Thanks the in silico experiment the node acting as a coordinator for the signaling cascades of different subcellular districts was identified. Then, the consequences on cellular function of its inhibition were experimentally evaluated in vitro. This goal was targeted by representing the biochemical machinery that drives the capacitation as a biological network that, on the basis of its topological proprieties, was considered as a scale free network. In fact, the node degree (or connectivity), *k*, which indicates how many links the node has to other nodes, followed a power-law distribution of the number of links per node and, in parallel, the tendency to develop clusters of nodes, the so called clustering coefficient *C*I [*C*I = 2*n*I/*k*(*k*-1), where *n*I is the number of links connecting the *k*I neighbors of node I to each other] was independent of the number of links per node. This architecture conferred to the capacitation network high signaling efficiency and robustness against random failure [5] and, in keeping with the Barabási-Albert model [8], made evident that a relatively small number of nodes (the hubs) were highly connected and most of the nodes were scarcely linked. Thus, it was possible to identify the hubs, i.e. the molecules playing a key role in post-ejaculatory maturation of spermatozoa and to localize them in different subcellular districts. In addition, the functional meaning and the localization of all the molecules involved in capacitation were studied and the nodes that interconnect the intracellular compartments were identified. It is really impressive that these nodes were only three: [Ca^2+^]_i_, ATP and "actin polymerization". More in detail [Ca^2+^]_i _and ATP are linked with a high number of nodes (28 and 15 respectively) and exerted the function of ubiquitous second messengers ([Ca^2+^]_i_) or of metabolic sustain (ATP). It is important to note that it has been recently demonstrated that their ablation from the network causes the collapse of network structures [5]. On the contrary, the "actin polymerization" bounds in a specific way, having only 8 links, all the subcellular compartments involved in capacitation (cytosol, cytoskeleton, mitochondria and acrosome). It is suggestive that it does not link the nucleus, the only compartment that remains stable until fertilization.

In particular it links:

- phosphatidic acid (i.e. the product of PLD activity): it is known that actin polymerization depends on PLD activation, which occurs via the HCO3 2/cAMP/PKA pathway or via the G-protein coupled receptor (GPCR) (LPA-receptor)/PKC pathway [9];

- ATP: the actin is an ATPase and its polymerization depends on ATP cellular concentration, in addition, the ATP is the most important molecule of spermatozoa energy metabolism. In fact, the metabolic energy production in spermatozoa depends on glycolysis and/or on mitochondrial oxidative [10];

- F-actin and G-actin: for recent reviews on actin polymerization see [11-13];

- [Ca^2+^]_i_: the intracellular calcium is one of the most important second messengers that drives the intracellular signaling during capacitation;

- OAM and PM fusion: see below for explanation;

- Protein synthesis: the incubation with the mitochondrial translation inhibitor D-chloramphenicol almost completely blocked spermatozoa actin polymerization [6].

In this context it is important to note that the links of actin polymerization involve three of the most linked nodes found in capacitation (ATP, [Ca^2+^]_i _and PLD) and that this node was *per se *one of the most linked node. Thus, it is possible to hypothesize that a message involving the actin polymerization node could reach all the cell districts, perturbing the whole signal transduction system of spermatozoa.

In addition, it has been found that the removal of "actin polymerization" node caused an important effect: the main topological parameters of the capacitation network were virtually unaffected but some nodes remained out from the network: OAM fusion, PM fusion, G-actin, F-actin, mitochondrial protein translation. This finding allows to predict that the functional ablation of actin polymerization during the in vitro capacitation could cause the spermatozoa inability to undergo AR (the OAM and PM fusion did not take place) while, the different signalling pathways remained unaffected.

To obtain an experimental confirmation of the model-based prediction, the actin polymerization was inhibited, as clearly confirmed by confocal analyses, by the incubation of spermatozoa under capacitating condition in the presence of CD.

The effect of this drug on the whole cell function was immediately evident: the treated spermatozoa lost the ability to undergo AR when exposed to the physiological agonist, the sZP. In parallel, the biochemical analyses showed that the main signaling pathways involved in capacitation were unaffected by the treatment. Particularly the capacitation implies a marked calcium-dependent rearrangement of membrane structure that, in turn, determines the increase of the plasma membrane (PM) and of the outer acrosome membrane (OAM) fusogenicity. This event resulted to be unaffected by CD administration as demonstrated by CTC staining.

Another important signaling pathway involves the protein tyrosine phosphorylation, via sAC/cAMP/PKA. It is known that the protein tyrosine phosphorylation pattern of spermatozoa changes during the capacitation in several species such as human, mice, cattle, pig, hamster and cat [14-16]. In boar spermatozoa a typical protein phosphorylation pattern has been identified [17], and the CD treatment did not exert any detectable effect on this parameter.

The PLC-γ1 plays a key role in coupling actin cytoskeleton and membrane dynamics to the calcium metabolism. When activated this enzyme migrates from the cytosol to the membrane [18] where it hydrolyses the phosphatidylinositol 4,5-bisphosphate [PI(4,5)P2] generating inositol 1,4,5-trisphosphate, a universal calcium-mobilizing second messenger, and diacylglycerol, an activator of protein kinase C. the present results showed that the PLC-γ1 translocation was unaffected by the presence of CD in the capacitation medium.

Ultimately, the effect of spermatozoa incubation under capacitating condition in presence of CD on calcium metabolism was tested. In fact, the spermatozoa signaling machinery strictly depends on intracellular calcium concentration [19-21]. During the capacitation the Ca^2+ ^behaves as a second messenger converting extracelluar stimuli in chemical response involving a myriad of molecular system, such as, protein kinase C (PKC), protein kynase C (PKA), actin, and many others. The completion of capacitation enables the spermatozoa to respond to the interaction with oocyte zona pellucida with a very fast surge of [Ca^2+^]_I_, during AR. In the present work the incubation of spermatozoa in presence of CD did not exert any detectable effect on this event.

From these findings it is evident that the spermatozoa inability to fulfill the physiological goal, the ZP-induced AR, is paralleled by the maintained efficiency of the main signaling pathways, in agreement with the computational model-based prediction.

The data until now available attribute to the actin dynamics a structural role during capacitation and AR. In particular, it is known that gradually the PM acquires the ability to fuse with the OAM thanks to the remodelling of its lipid composition and architecture [9,19,22-24]. The developing network of actin acts as a diaphragm between the two membranes avoiding their fusion. Once the capacitation was completely achieved and the physiological stimulus (ZP proteins) was detected, the AR takes place and the ZP-induced calcium peak causes the fast depolymerisation of actin structure. If this model was correct it was reasonably to expect that the block of actin polymerization (i.e. the treatment of spermatozoa with CD) in early phases of capacitation leads the increase in the percentage of spermatozoa showing the loss of acrosome integrity due to the inefficiency of separation of PM and OAM. In our experiments, on the contrary, it was found that, even in the absence of the actin network, the PM and OAM did not fuse. Thus, the results of the present work could complete and revamp the current concept, leading us to hypothesize that in swine spermatozoa, as demonstrated in other cells, the role of actin cytoskeleton overcomes this merely structural function. It is possible to conceive that, linking all the cellular districts involved in capacitation, the actin polymerization could have a role of general coordination of this pivotal biological event. In particular, it is noteworthy that this node links three hubs of the system, thus, strengthening the hypothesis that actin polymerization acts as an important node of coordination trough the information flow. This supposition is in keeping with the newly emerging evidence that in different cellular systems the cytoskeleton is not only a mechanical support for the cell, but exerts a key role in signaling. In fact, it was proposed that "independent of its mechanical strength, the filaments of the cytoskeleton form a continuous, dynamic connection between nearly all cellular structures, and they present an enormous surface area on which proteins and other cytoplasmic components can dock" [25]. This is strengthened by the finding that the plasma membrane surface area of a 20-μm-diameter generic cell is on the order of 700 μm^2^, in contrast, the total surface area of a typical concentration of 10 mg/ml F-actin is 47,000 μm^2 ^[25] and that the diffusion along cytoskeletal tracks could be a reliable alternative to other established ways of intracellular trafficking and signaling, and could therefore provide an additional level of cell function regulation [26]. One implication of this role is that, together with other well known molecules involved in intracellular signaling, such as Ca^2+ ^or ATP, the actin cytoskeleton might provide a signal transduction route and macromolecular scaffold, which, during eukaryotic evolution, contributes to the spatial organization of signaling pathways components [27].

## Conclusions

From these data it is possible to take some considerations:

1. it is possible to speculate that the male germ cell representation as a biological network could be an important tool, because it would allow to study not only the molecular components of these cells, but also their intracellular localization and intermolecular interactions in signaling pathways. In this context it is important to note that in complex systems the whole system is more than the sum of its single components;

2. the present findings could be discussed as the evidence that spermatozoa behave as complex systems. In this optic the acquisition of the fertilizing ability is a system propriety that emerges when these cells are considered in their whole signaling network. If the single subcellular element is unperturbed by an external factor, but the coordination among all of them is broken, the male gamete becomes unable to express the ability to physiologically respond to the stimulus (the sZP). In addition, the molecule/s acting as controller of cross-talking among the different intracellular compartments could become a sort of "Achilles' heel" of cellular signaling machinery, when compromised.

3. it was already known that the actin cytoskeleton is a highly dynamic structure involved in capacitation. From the present data it emerges that actin polymerization could be involved in coordination of signaling during the capacitation.

In our opinion these findings strength the usefulness of the combined use of a computational and an experimental approach, could have important implications in diagnostics and therapeutics of male infertility, as well in contraceptive strategies, and could contribute to the knowledge of the role played by cytoskeleton in cell signal transduction.

## Methods

### Chemicals

Chemicals were of the purest analytical grade. Fetal Calf Serum (FCS) was purchased from GIBCO BRL (Milano, ITALY). Fluo-3-AM, Pluronic F-127, propidium iodide (PI), Hoecst 33258 were from Calbiochem, (San Diego, CA); Percoll, Pisum sativum agglutinin-FITC (fluorescein isothiocyanate) conjugated (FITC-PSA), Dulbecco with Ca^++ ^and Mg^++^, TCM199, bovine serum albumine (BSA), sodium pyruvate, glucose, calcium lactate and all others chemicals were from Sigma Chemical Co. (St. Louis, MO).

### Capacitation computational model

The biological network representing the molecular events occurring during the capacitation of boar spermatozoa was realized in accordance with Bernabò et al. [5], introducing some specie-specific modification, using Cytoscape 2.6.3 software (http://www.cytoscape.org). The representation of subcellular compartments was made with the Cytoscape plugin Cerebral v.2 (http://www.pathogenomics.ca/cerebral/).

To evaluate the effect of the removal of the "actin polymerization" node from the capacitation network a new network was realized omitting this node ("no actin polymerization network"). This network was spatially represented using the Cytoscape Spring-embedded Layout. This program is based on a force-directed paradigm. Network nodes are treated like physical objects that repel each other, such as electrons. The connections between nodes are treated like metal springs attached to the pair of nodes. These springs repel or attract their end points according to a force function. The layout algorithm sets the positions of the nodes in a way that minimizes the sum of forces in the network" [15].

The statistical and topological analyses of the networks were carried out, considering the network as undirected, by the Cytoscape plugin Network Analyzer (http://med.bioinf.mpi-inf.mpg.de/netanalyzer/).

### Spermatozoa preparation

Semen samples were collected and processed by an already validated protocol [28,29]. The incubation under capacitating condition was carried out in TCM199 medium added with 13.9 mM glucose, 1.25 mM sodium pyruvate, 2.25 mM calcium lactate and 100 μg/ml kanamycin (300 mOsm/kg, pH 7.4) at a final concentration of 1 × 10^8 ^cells/ml for 4 h at 38.5°C in 5% CO_2 _humidified atmosphere (Heraeus, Hera Cell) for 4 h. Only the samples maintaining a mean viability, assessed as previously described (Barboni et al. 1995), of at least 80% at the end of the culture were considered for the following analysis. The sperm samples were processed as freshly ejaculated (T0), incubated under control conditions (CTR) or were constantly maintained in the presence of 20 μM of citochalasin D (CD).

In addition, in order to verify the effect of CD treatment on the rate of actin polymerizaton the sperm samples (T0, CTR and CD) were stained, as described by Bernabò et al. [30], with TRITC-conjugated phalloidin, a molecule exhibiting an high binding affinity for F-actin. For each treatment, at least 100 spermatozoa were observed by using the confocal microscope (Radiance 2000, BioRad, UK).

### Detection of ZP-induced AR

The competence of in vitro incubated spermatozoa to undergo AR in response to solubilised zonae pellucidae (ZP) co-incubation was further evaluated as a functional endpoint of the capacitative state. To this aim aliquots of spermatozoa were incubated under different experimental conditions, and after the incubation period were exposed for 30 min to solubilised ZP (sZP) (10 ZP/μl), obtained as reported [29,31]. The percentage of sZP-induced AR was detected by FITC-PSA and Hoechst staining [29,31]. Since spermatozoa incubated for in vitro capacitation spontaneously and variably exocytate their acrosomal content in the absence of any specific stimulus, the acrosomal exocytosis before exposing spermatozoa to ZP was considered as "spontaneous AR". This spontaneous AR was subtracted from total AR, in order to obtain the incidence of the true sZP-induced AR, in keeping with Bernabò et al. [30].

### CTC staining

The chlortetracycline stain (CTC) was used to evaluate the completion of calcium-dependent membrane remodelling, as previously described (Mattioli et al., 1996; Barboni, 1994). In detail, 10 μl for each sperm sample incubated under different conditions (CTR or CD) were stained, on a warm stage, with 10 μl CTC 750 μM/L (solubilized in TRIS-HCl 20 mM; NaCl 130 mM; L-cisteina 5 mM, pH 7.8); after 30 sec 10 μl glutaraldehyde 1% and10 μl mounting medium were added. For each sample were assessed at least 200 spermatozoa and the percentage of spermatozoa displaying fluorescence pattern C indicative of capacitation (CTC fluorescence over the post acrosomal area) was calculated.

### Biochemical evaluation of tyrosine phosphorilation pattern and PLCγ-1 relocalization

Samples of spermatozoa were centrifuged (500 × g for 3 min.), washed in 1 ml of Phosphate Buffer Saline (PBS) pH 7.5 and then resuspended in following lysis buffer (Buffer-A): 50 mM Tris-HCL pH 7.5, 150 mM NaCl, 2 mM EDTA pH 8, 1 mM Phenyl Methyl Mulphonyl Fluoride (PMSF), 1 mM Sodium Orthovanadate (Na_3_VO_4_), 10 mM Sodium Fluoride, 10 ug/ml Leupeptin, 10 ug/ml Antipain, 100 units/ml Aprotinin. After 20 strokes in a Dounce homogenizer, aliquots of total extracts were stored at -80°C until use and the remaining homogenated samples were gently extracted 30 min on ice, and then centrifugated at 35.000 × g for 35 min at 4°C in Ultracentrifuge (Beckman-OPTIMA™). The supernatant (cytosolic fractions) was precipitated with 8 volumes of Acetone while the pellet (membrane fraction) extracted in Buffer A containing 0,1% Triton X 100 as described above. After centrifugation at 5.000 rpm in Sorvall-SW41 rotor, the cytosolic and membrane protein pellets were resuspended in buffer containing 2% SDS with protease and phosphatase inhibitors. An aliquot of total lysates, cytosolic and membrane fractions were used to evaluate the amount of proteins [32].

Equal amounts of sperm's total, cytosolic and membrane proteins (50-100 μg/lane) were separated by 8%-10%SDS-PAGE and then transferred onto nitrocellulose (Hybon C Extra, Amersham Bioscience) following standard procedures. Brain proteins were used as positive control The membrane was blocked for 1 hour at room temperature with Tris Buffer Saline (TBS: 20 mM Tris pH 7.6, 150 mM) plus 3% (w/v) non fat dry milk and immunoblottings were performed with the following antibodies: 1:200 dilution of anti- PLC-γ1 (Santa Cruz), 1:4000 dilution of anti-Phosphotyrosine antiboby clone 4G10 (UPSTATE) or anti-alpha Tubulin antiboby clone B-5-1-2 (SIGMA) for 2 hours at room temperature in TBS containing 1% (w/v) BSA and 0.05% (v/v) Tween 20. After incubation, the membrane was washed four times for 5 min. with TBS containing 0.05% (v/v) Tween 20 and then incubated for 1 hour at room temperature with 1:4000 dilution of goat anti mouse or anti-rabbit IgG horseradish peroxidase (HRP) conjugated (Santa Cruz) in TBS containing 1% (w/v) non fat dry milk and 0.05% (v/v) Tween 20. The signal was developed by ECL detection system (Amersham Bioscience). When necessary, the nitrocellulose membranes were stripped with the following buffer (62,5 mM Tris HCL pH 6.8, 2% (w/v) SDS, 100 mM 2-mercapto-ethanol for 45 min at 60 C° with constant shacking. The membranes were then extensively PBS washed and then reprobed as described.

### Sperm pre-embedding electron microscopic immunocytochemistry

Phospholipase Cγ1 (PLC-γ1) localization was performed on T0, and on spermatozoa incubated under capacitating conditions with or without CD (CTR and CD samples). All different groups of spermatozoa were collected and immediately fixed in 2% paraformaldehyde and 0.5% glutaraldehyde in 0,1 M cacodylate buffer PH 7.4 for 1 h at 4°C. After washing spermatozoa in PBS/1% BSA/0.05% Tween 20 by two centrifugations at 800 g for 5 min., samples were resospended in Goat Serum (Sigma) 1:5 PBS/1% BSA for 1 h at room temperature, and then incubated in a rabbit anti - PLC-γ1 (Santa Cruz) 1:100 in PBS/1% BSA overnight at 4°C. The anti - PLC-γ1 was then visualized with an anti-rabbit IgG - Gold colloidal Particle - 10 nm (EY Laboratories, Inc.) 1:20 in PBS/1% BSA for 1 h at room temperature. After several washes in PBS/1% BSA/0.05% Tween 20 spermatozoa were washed in 0.1 M cacodylate buffer PH 7.4 fixed with 2% paraformaldehyde and 0.5% glutaraldehyde in 0.1 M cacodylate buffer PH 7.4 for 1 h at 4°C and postfixed with 1% osmium tetroxide in 0.1 M cacodylate buffer PH 7.4 for 30 min. at 4°C. The samples were then dehydrated with a graded series of ethanol and embedded in LR White resin (Polyscience, Inc.). Ultrathin sections were stained with uranyl acetate and lead citrate before being examined in a Zeiss transmission electron microscope (EM900).

### ZP-induced intracellular calcium rise

The calcium probe fluo-3-AM was used to assess the variations in the intracellular calcium concentration in response to the sZP coincubation. To this aim a 2 mM stock solution of fluo-3-AM was solubilized in dry dimethylsulphoxyde, containing 37.5 g/l Pluronic F-127. Spermatozoa (5 × 10^6 ^spermatozoa/ml final concentration) were incubated at 38.5°C for 30 min in Dulbecco's phosphate buffer with Ca^2+ ^and Mg^2+ ^containing 4 μM fluo-3-AM avoiding the exposition of samples to the light. The samples were, then, putted in a warmed chamber containing the spermatozoa suspended in Dulbecco's phosphate buffer with Ca^2+ ^and Mg^2+ ^and observed under confocal microscopy (Radiance 2000, BioRad, UK) for 3 - 4 min. The first 60 - 100 sec. of examination were carried out to exclude photobleaching. When the sample fluorescence emission was stable, an aliquot of sZP was added and the observation was performed for 3-5 min. To confirm the responsiveness of the system, the not responding samples were exposed to 10 μM A23187 to assess their ability to respond to the calcium ionophore-induced entry. Only responding spermatozoa were considered as functionally integer. All the samples were assessed using the same exposition parameters to avoid artefacts in the data analysis. The fluorescence emission, expressed as Fluorescence Arbitrary Units, was proportional to the intracellular calcium concentration.

### Image analysis

The acquisition of images by confocal microscopy was carried out using Lasersharp 2000 software (Biorad, UK). The image analysis was realized by LaserPix 4.0 (Biorad, UK).

### Statistical analysis

Data reported in this paper are expressed as mean ± standard deviation of three independent experiments, each performed in duplicate. The data were checked for normal distribution (Shapiro-Wilks W test) and then compared by ANOVA test (StatistiKL Version β). The differences were considered significant and highly significant for p values of <0.05 and <0.01, respectively.

## Authors' contributions

NB have made substantial contributions to conception and design of the work and carried out the data analysis; AM performed the protein electrophoresis experiments; VR performed the TEM experiments; PL carried out the in vitro experiments; PB, MM and BB participated to the experimental design and have been involved in revising the manuscript critically for important intellectual content. All Authors read and approved the final manuscript.

## References

[B1] Knoll AH Javaux EJ Hewitt D Cohen P Eukaryotic organisms in Proterozoic oceans Phil Trans R Soc B 2006 361 1023 1038 10.1098/rstb.2006.1843 16754612 PMC1578724

[B2] Weng G Bhalla US Iyengar R Complexity in biological signaling systems Science 1999 284 92 95 10.1126/science.284.5411.92 10102825 PMC3773983

[B3] Barabási AL Oltvai ZN Network biology: understanding the cell's functional organization Nature Reviews 2004 5 101 113 14735121 10.1038/nrg1272

[B4] Albert Scale-free networks in cell biology J Cell Sci 2005 118 4947 4957 10.1242/jcs.02714 16254242

[B5] Bernabò N Mattioli M Barboni B The spermatozoa caught in the net: the biological networks to study the male gametes post-ejaculatory life BMC Syst Biol 2010 4 87 20565893 10.1186/1752-0509-4-87 PMC2905340

[B6] Gur Y Breitbart H Mammalian sperm translate nuclear-encoded proteins by mitochondrial-type ribosomes Genes Dev 2006 20 411 416 10.1101/gad.367606 16449571 PMC1369042

[B7] Mattioli M Barboni B Lucidi P Seren E Identification of capacitation in boar spermatozoa by chlortetracycline staining Theriogenology 1996 45 373 381 10.1016/0093-691X(96)81099-5 16727801

[B8] Barabási AL Albert R Emergence of scaling in random networks Science 1999 286 509 512 10521342 10.1126/science.286.5439.509

[B9] Breitbart H Cohen G Rubinstein S Role of actin cytoskeleton in mammalian sperm capacitation and the acrosome reaction Reproduction 2005 129 263 268 10.1530/rep.1.00269 15749953

[B10] Storey BT Mammalian sperm metabolism: oxygen and sugar, friend and foe Int J Dev Biol 2008 52 427 437 10.1387/ijdb.072522bs 18649255

[B11] Dominguez R Actin filament nucleation and elongation factors--structure-function relationships Crit Rev Biochem Mol Biol 2009 44 351 66 10.3109/10409230903277340 19874150 PMC2787778

[B12] Campellone KG Welch MD A nucleator arms race: cellular control of actin assembly Nat Rev Mol Cell Biol 2010 11 237 51 10.1038/nrm2867 20237478 PMC2929822

[B13] Carlsson AE Actin dynamics: from nanoscale to microscale Annu Rev Biophys 2010 39 91 110 10.1146/annurev.biophys.093008.131207 20462375 PMC2967719

[B14] Visconti PE Bailey JL Moore GD Pan D Olds-Clarke P Kopf GS Capacitation of mouse spermatozoa. I. Correlation between the capacitation state and protein tyrosine phosphorylation Development 1995 121 1129 1137 7743926 10.1242/dev.121.4.1129

[B15] Urner F Sakkas D Protein phosphorylation in mammalian spermatozoa Reproduction 2003 125 17 26 10.1530/rep.0.1250017 12622692

[B16] Barbonetti A Vassallo MR Cinque B Antonangelo C Sciarretta F Santucci R D'Angeli A Francavilla S Francavilla F Dynamics of the global tyrosine phosphorylation during capacitation and acquisition of the ability to fuse with oocytes in human spermatozoa Biol Reprod 2008 79 649 656 10.1095/biolreprod.108.068254 18562705

[B17] Bravo MM Aparicio IM Garcia-Herreros M Gil MC Peña FJ Garcia-Marin LJ Changes in tyrosine phosphorylation associated with true capacitation and capacitation-like state in boar spermatozoa Mol Reprod Dev 2005 71 88 96 10.1002/mrd.20286 15736131

[B18] Spungin B Breitbart H Calcium mobilization and influx during sperm exocytosis J Cell Sci 1996 109 1947 1955 8832417 10.1242/jcs.109.7.1947

[B19] Breitbart H Signaling pathways in sperm capacitation and acrosome reaction Cell Mol Biol 2003 49 321 327 12887084

[B20] Tulsiani DR Zeng HT Abou-Haila A Biology of sperm capacitation: evidence for multiple signalling pathways Soc Reprod Fertil Suppl 2007 63 257 272 17566278

[B21] Abou-haila A Tulsiani DR Signal transduction pathways that regulate sperm capacitation and the acrosome reaction Arch Biochem Biophys 2009 485 72 81 10.1016/j.abb.2009.02.003 19217882

[B22] Oikonomopoulou I Patel H Watson PF Chantler PD Relocation of myosin and actin, kinesin and tubulin in the acrosome reaction of bovine spermatozoa Reprod Fertil Dev 2009 21 364 377 10.1071/RD08166 19210928

[B23] Correa LM Thomas A Meyers SA The macaque sperm actin cytoskeleton reorganizes in response to osmotic stress and contributes to morphological defects and decreased motility Biol Reprod 2007 77 942 953 10.1095/biolreprod.107.060533 17823088

[B24] Cohen G Rubinstein S Gur Y Breitbart H Crosstalk between protein kinase A and C regulates phospholipase D and F-actin formation during sperm capacitation Dev Biol 2004 267 230 41 10.1016/j.ydbio.2003.10.034 14975729

[B25] Janmey PA The cytoskeleton and cell signaling: component localization and mechanical coupling Physiol Rev 1998 78 763 778 9674694 10.1152/physrev.1998.78.3.763

[B26] Shafrir Y ben-Avraham D Forgacs G Trafficking and signaling through the cytoskeleton: a specific mechanism J Cell Sci 2000 113 2747 2757 10893190 10.1242/jcs.113.15.2747

[B27] Forgacs G Yook SH Janmey PA Jeong H Burd CG Role of the cytoskeleton in signaling networks J Cell Sci 2004 117 2769 2775 10.1242/jcs.01122 15150320

[B28] Bernabò N Tettamanti E Pistilli MG Nardinocchi D Berardinelli P Mattioli M Barboni B Effects of 50 Hz extremely low frequency magnetic field on the morphology and function of boar spermatozoa capacitated in vitro Theriogenology 2007 67 801 815 17196643 10.1016/j.theriogenology.2006.10.014

[B29] Bernabò N Tettamanti E Russo V Martelli A Turriani M Mattioli M Barboni B Extremely low frequency electromagnetic field exposure affects fertilization outcome in swine animal model Theriogenology 2010 73 1293 1305 20176397 10.1016/j.theriogenology.2009.12.010

[B30] Bernabò N Pistilli MG Mattioli M Barboni B Role of TRPV1 channels in boar spermatozoa acquisition of fertilizing ability Mol Cell Endocrinol 2010 323 224 231 20219627 10.1016/j.mce.2010.02.025

[B31] Barboni B Mattioli M Seren E Influence of progesterone on boar sperm capacitation J Endocrinol 1995 144 1 8 10.1677/joe.0.1440013 7891014

[B32] Lowry OH Rosebrough NJ Farr AL Randall RJ Protein measurement with the Folin phenol reagent J Biol Chem 1951 193 265 275 14907713

